# Organizing healthcare delivery in the era of disease-modifying therapies for Alzheimer’s Disease: an Italian RAND/UCLA consensus

**DOI:** 10.1007/s10072-026-09452-z

**Published:** 2026-10-09

**Authors:** Alessandro Padovani, Mattia Altini, Giuseppe Bellelli, Marco Bozzali, Ovidio Brignoli, Annachiara Cagnin, Antonio Cherubini, Manuele Iannacci, Raffaele Lodi, Giancarlo Logroscino, Camillo Marra, Carlo Nicora, Simone Parretti, Anna Sacco, Patrizia Spadin, Mario Zappia

**Affiliations:** 1 Department Clinical and Experimental Sciences, University of Brescia, Brescia, Italy; 2 Department of Continuity of Care and Frailty, ASST Spedali Civili Di Brescia, Brescia, Italy; 3 Local Health Authority of Modena (AUSL Modena), Modena, Italy; 4 Italian Society of Leadership and Management in Medicine (SIMM), Meldola, Italy; 5 School of Medicine and Surgery, University of Milano-Bicocca, Milan, Italy; 6 Acute Geriatric Unit, IRCCS Foundation San Gerardo, Monza, Italy; 7 Department of Neuroscience, Rita Levi-Montalcini, University of Torino, Turin, Italy; 8 Neurology Unit 2U, Azienda Ospedaliero-Universitaria Città Della Salute E Della Scienza Di Torino, Turin, Italy; 9 Italian College of General Practitioners and Primary Care Professionals (SIMG), Florence, Italy; 10 Department of Neuroscience, University of Padova, Padua, Italy; 11 Padua Neuroscience Centre, University of Padova, Padua, Italy; 12 Geriatric Admission Unit and Research Centre On Aging, IRCCS INRCA, Ancona, Italy; 13 Department of Clinical and Molecular Sciences (DISCLIMO), Università Politecnica Delle Marche, Ancona, Italy; 14 Functional and Molecular Neuroimaging Unit, IRCCS Institute of Neurological Sciences of Bologna, Bologna, Italy; 15 Department of Biomedical and Neuromotor Sciences (DIBINEM), University of Bologna, Bologna, Italy; 16 Centre for Neurodegenerative Diseases and the Aging Brain, Department of Clinical Research in Neurology, University of Bari ‘Aldo Moro’, Tricase, Lecce, Italy; 17 Department of Translational Biomedicine and Neuroscience (DiBraiN), University of Bari, Bari, Italy; 18 Department of Psychology, Catholic University of the Sacred Heart, Milan, Italy; 19 Fondazione Molina Varese, Varese, Italy; 20 IQVIA, Milan, Italy; 21 Italian Alzheimer’s Disease Association (AIMA), Milan, Italy; 22 Italian Society of Neurology (SIN), Siena, Italy; 23 Department of “G.F. Ingrassia”, University of Catania, Catania, Italy

**Keywords:** Alzheimer’s disease, Disease-modifying therapies, Care pathways, Healthcare organization, Consensus study, Italy

## Abstract

**Background:**

Alzheimer’s disease (AD) poses a growing clinical and organizational challenge, requiring coordinated, multidisciplinary care models. In Italy, significant regional variability in resources and access to diagnostic services persists, while the implementation of community-based care models, the expanding role of biomarkers, and the advent of disease-modifying therapies (DMTs) further highlight the need for a more coordinated and equitable dementia care network.

**Objective:**

Develop system-level recommendations to optimise the organisation of AD care delivery in Italy in anticipation of DMTs introduction.

**Methods:**

Using the RAND/UCLA Appropriateness Method, a multidisciplinary panel rated 55 statements across nine domains, evaluating appropriateness in two rounds and priority of implementation in a third round.

**Results:**

Consensus was achieved for all statements, with high appropriateness and priority ratings. The statements outline a regional, network-based model organised across territorial services, Spoke and Hub centres, with formal governance responsible for pathway definition and monitoring. Referral pathways from primary care are based on cognitive and frailty assessment, with non-frail patients referred to Spoke for clinical, neuropsychological and neuroradiological evaluation. Patients potentially eligible for DMTs are referred to Hub centres, where diagnostic confirmation is completed and multidisciplinary teams assess treatment eligibility. When authorised, Hub centres are responsible for DMTs prescription and administration. The statements emphasise the need for standardised neuropsychological and neuroradiological protocols for amyloid-related imaging abnormalities (ARIA) monitoring, alongside the progressive adoption of plasma biomarkers, telemedicine, and structured communication with patients and caregivers.

**Conclusion:**

This consensus provides a pragmatic framework to support the reorganisation of AD care in Italy, ensuring that every patient is directed to the most appropriate level of care across a flexible regional network.

**Supplementary Information:**

The online version contains supplementary material available at https://doi.org/10.1007/s10072-026-09452-z.

## Introduction

Alzheimer’s disease (AD) is a chronic, progressive neurodegenerative disorder characterised by cognitive decline, prevalent memory deficit, functional impairment and behavioural disturbances [1, 2]. It represents the leading cause of dementia globally, accounting for approximately 60–80% of all cases [2].

According to Alzheimer Europe, approximately 9 million people were living with dementia in Europe in 2025 [3]. In Italy, the number of individuals affected by dementia in the same year was estimated at 1,436,859, representing around 2.43% of the national population, of whom an estimated 600,000 are living with AD [3, 4]. Italy’s demographic profile, characterised by one of the oldest populations globally, further amplifies the projected rise in disease prevalence, with the number of people living with AD expected to increase by approximately 50% over the next 30 years [5]. Beyond its increasing prevalence, AD imposes a substantial economic burden in Italy, with total annual costs estimated at approximately €15,6 billion, approximately 80% of which is borne directly by families [6, 7]. Therefore, AD represents not only a neurological disorder with a strong clinical burden but also a condition with extensive socio-economic implications.

For many years, therapeutic strategies for AD were predominantly symptomatic. Cholinesterase inhibitors along with the N-methyl-D-aspartate (NMDA) receptor antagonist demonstrated only transient symptomatic benefit but did not alter the underlying disease trajectory [8].

The development of monoclonal antibodies targeting aggregated amyloid species has subsequently reshaped the therapeutic landscape of AD. In 2025, the European Medicines Agency (EMA) granted marketing authorisation for two of these disease-modifying therapies (DMTs) for early Alzheimer’s disease, marking a significant milestone in the evolution of AD treatment [9, 10]. These DMTs demonstrated consistent and clinically relevant slowing of disease progression across multiple endpoints, targeting the underlying pathophysiology of AD with the potential to alter its clinical trajectory [11]. Healthcare systems must reorganise care pathways to ensure timely diagnosis, biomarker-guided patient selection, and structured monitoring including Magnetic Resonance Imaging (MRI) surveillance for amyloid-related imaging abnormalities (ARIA) [12, 13].

In Italy, the evolution of dementia care is being shaped not only by therapeutic innovation, but also by the ongoing implementation of community-based care models under Ministerial Decree 77/2022 (DM77) and the increasing availability of advanced diagnostic procedures and biomarkers [14]. Taken together, these factors create an opportunity to redesign the AD care network while simultaneously placing new demands on the system in terms of early diagnosis, biomarker-guided patient selection, and longitudinal follow-up.

AD care in Italy has evolved through the establishment of dedicated services, initially organised as Alzheimer’s Evaluation Units (UVA) and in 2014 restructured as Centres for Cognitive Disorders and Dementias (CCDDs). Currently, a total of 536 CCDDs operate nationwide as publicly funded services within the National Health Service [15]. These centres are authorised to prescribe anti-dementia and antipsychotic medications for individuals with dementia and provide a range of psychosocial, educational, and rehabilitative interventions, including post-diagnostic support for patients and caregivers [16].

Although CCDDs constitute the backbone of AD’s diagnostic and management pathways, substantial regional variability persists in terms of available resources, multidisciplinary integration, and access to advanced diagnostic technologies. Evidence indicates marked differences in the availability of Positron Emission Tomography (PET), Cerebrospinal Fluid (CSF) or plasma biomarker testing and neuropsychological assessments with centres in Northern Italy demonstrating greater access compared with those in Central and Southern regions, including the islands [16].

Such disparities risk widening rather than narrowing if the Italian care network is not reimagined. Within this context, the RAPID project (*Revolutionizing Alzheimer’s Patient care model. Initiatives Driving the future*) developed, through structured multi-stakeholder consensus, pragmatic recommendations for reorganising AD care in Italy to meet the demands of both routine clinical management and the introduction of novel disease-modifying therapies.

## Methods

### Study design

The RAPID project aimed to develop pragmatic recommendations for defining the optimal organisation of AD care delivery in Italy in anticipation of the introduction of monoclonal antibodies (mAbs) therapies. To obtain structured expert consensus on the appropriateness and priority of implementation of key organisational components, healthcare interventions, and clinical decisions within the AD care pathway, the project adopted the RAND/UCLA Appropriateness Method (RAM), an internationally validated consensus methodology [17].

The project was conducted with the support of Eisai. The consensus process was coordinated by the IQVIA Implementation Science (IS) team, comprising two consultants with expertise in implementation science research and consensus methodologies. They were responsible for the methodological setup and conduct of the consensus process, including statement drafting and refinement, panel management, and final analysis of results.

In line with RAM protocol, Eisai and IQVIA IS team did not participate in any voting rounds.

### Panel composition

The multidisciplinary panel comprised 13 experts, including six neurologists, two geriatricians, one neuroradiologist, one general practitioner (GP), two healthcare planning experts, and one representative from a patient association.

Panel members were identified by the IQVIA IS team based on their consolidated experience and national visibility, gained through participation in initiatives at national institutions and scientific societies, and in publications focused on the evolution and optimisation of AD patient pathways. To ensure representation of the diversity of care settings, the panel included clinicians from university hospitals, Scientific Institute for Research, Hospitalization and Care (IRCCS), and Level II-III hospitals and CCDDs, which were distributed across the entire national territory.

### Statements development and voting rounds

The IQVIA IS team conducted an initial narrative review of national and international intersocietal recommendations, AD care models from other countries, and the broader scientific literature [18–21]. Moreover, the team carried out a structured organisational assessment in seven Italian centres involved in the diagnosis and management of AD, located in different geographical areas of the country.

Based on semi-structured interviews with clinical and managerial staff, the assessment mapped current patient management pathways and the level of organisational readiness for the introduction of mAbs therapies.

The IQVIA IS team, following these activities, identified nine thematic domains representing the areas where pragmatic guidance is most needed to support reorganisation at both national and regional levels: network structure, primary care, the role of neurology, the role of neuropsychology, the role of neuroradiology, biomarkers, the role of geriatrics, organisation of infusion centres and DMT delivery, communication with patients and caregivers.

Panel members were appointed to lead one or more thematic domains according to their recognised expertise and current professional responsibilities within the AD care pathway. Discipline-specific domains were led by specialists directly involved in the corresponding area of clinical practice (e.g., the neuroradiology domain was led by the neuroradiologist). The two cross-cutting domains, network structure and organisation of infusion centres and DMT delivery, concern service design and governance and were therefore jointly led by healthcare planning experts and neurologists with documented experience in healthcare planning and the development of diagnostic and therapeutic care.

**Fig. 1:**
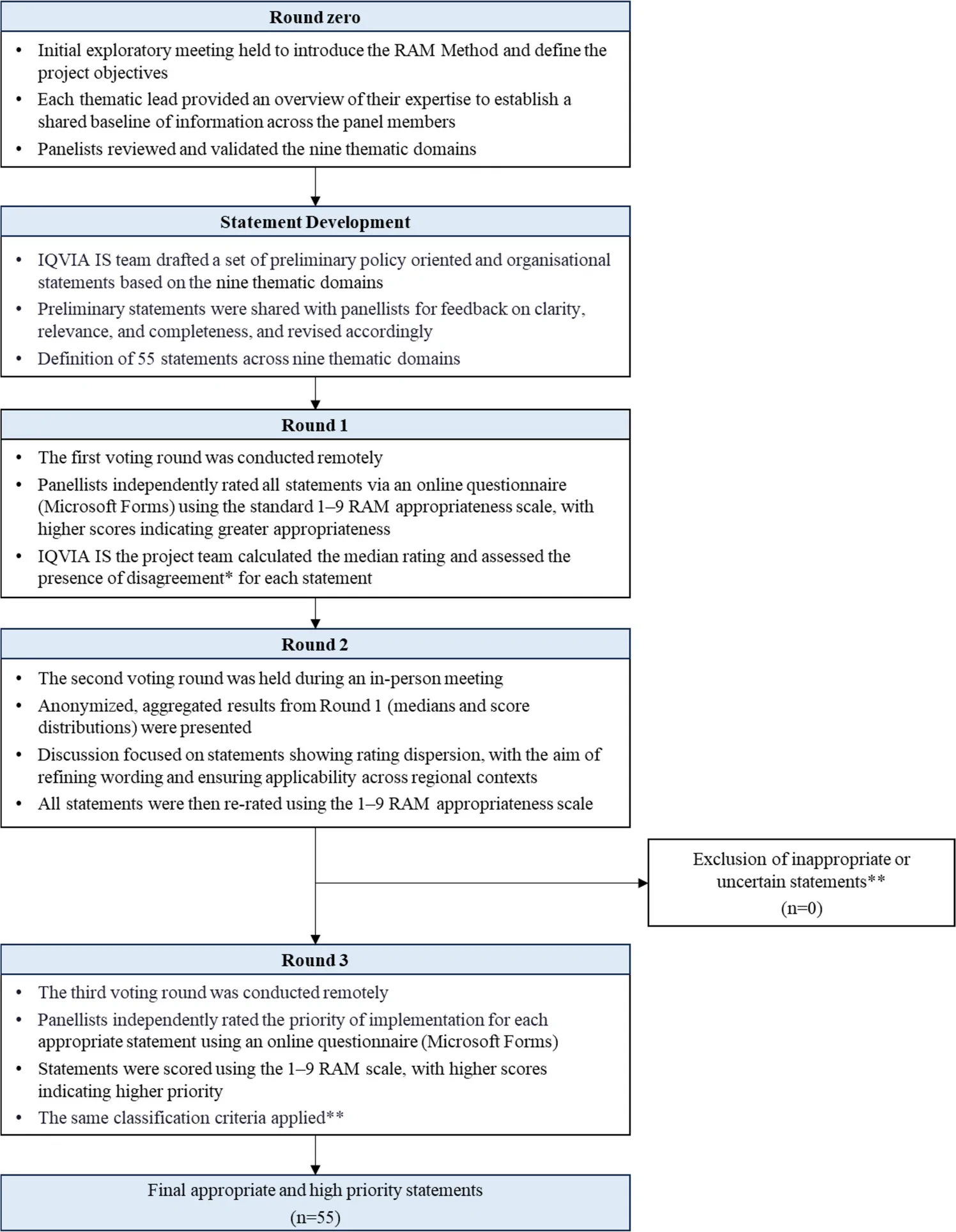
Overview of the consensus process, statement development and voting rounds. The figure illustrates the sequential steps followed to develop and vote the statements in accordance with the RAND/UCLA Appropriateness Method (RAM). *Disagreement was defined as a polarised distribution in which at least one-third of panellists rated the statement between 1 and 3 and at least one-third rated it between 7 and 9. **Statements were classified based on the combination of median rating and the presence or absence of disagreement: statements with a median of 7–9 and no disagreement were classified as appropriate (Round 1–2) or high priority (Round 3); those with a median of 1–3 and no disagreement were classified as inappropriate or low priority; and those with a median of 4–6 or any evidence of disagreement were classified as uncertain

Each domain lead presented an overview of the corresponding thematic domain to the full panel, and the domains were subsequently approved by all panellists. Based on this outcome, the IQVIA IS team drafted the preliminary set of statements for each domain. In accordance with the RAM protocol, these preliminary statements were circulated to the panel members to assess the content, wording, appropriateness and completeness of the statements and to propose additions, reformulations or deletions where relevant Fig. 1. Following this review, the statements were finalised by the IQVIA IS team, resulting in a set of 55 statements across the nine thematic domains, which were then submitted to the full panel for rating.

Conflicts of interest were assessed for all panel members prior to their involvement in the consensus process. The IQVIA IS team collected conflict of interest (COI) declarations from each panellist and reviewed them individually, evaluating both the nature and the extent of any disclosed interests in relation to the scope of the consensus statements. The disclosed relationships were judged not to pose a risk to the integrity of the consensus process, as the statements addressed organisational aspects of the AD care pathway rather than the endorsement of specific therapeutic interventions, which limited the extent to which such interests could influence statement development and voting.

The steps of the consensus process are detailed in Fig. 1. Statements were rated individually and anonymously by all panel members on the 9-point RAM scale, where 1 indicates the lowest and 9 the highest degree of appropriateness (Rounds 1–2) or of priority of implementation (Round 3).

In accordance with the RAM, classification was based on the panel median combined with the presence or absence of disagreement, defined as a polarised distribution in which at least one third of panellists rated a statement between 1 and 3 and at least one third between 7 and 9. Statements with a median of 7–9 and no disagreement were classified as appropriate (Rounds 1–2) or high priority (Round 3) and retained; those with a median of 4–6, or with evidence of disagreement, were classified as uncertain, discussed in plenary session, reformulated where necessary and re-rated in a subsequent round; those with a median of 1–3 and no disagreement would have been discarded.

## Results

### Statements overview

The 55 statements were grouped into nine thematic domains reflecting the key organisational components required to deliver high-quality, structured Alzheimer’s disease care in Italy, encompassing the full diagnostic and therapeutic pathway Tables 1 and 2.

**Table 1:** Network structure statements

Domain	Statement *N*	Statement
Newtork structure	1	Each Region organizes the diagnosis, treatment, and management of people with Alzheimer’s disease according to a regional network model, structured into Centres with different levels of expertise and functions along the care pathway
	2	Each Region appoints a dedicated governance body for the Alzheimer’s disease network, including a Network Coordinator and a multidisciplinary representation (neurology, geriatrics, neuroradiology, nuclear medicine, neuropsychology, and general practice)
	3	The main areas of responsibility of the regional governance body include: • definition and updating of the regional/network Integrated Care Pathway (ICP) and related Key Performance Indicators (KPIs) • definition and implementation of coordination mechanisms among the Centres within the network; • support to the definition and sizing of the Network based on epidemiological data and demand analysis; • monitoring of the indicators defined within the ICP
	4	Each regional network adopts a network Integrated Care Pathway (ICP) defining access criteria, roles, and timelines of the network nodes for diagnosis, treatment, and follow-up
	5	The regional network is structured into three functional levels: Hub Centres, Spoke Centres, and territorial services
	6	The regional network is embedded within the existing care system, which is already organized around Centres for Cognitive Disorders and Dementias (CCDDs). Within this framework, CCDDs may function as Hub or Spoke Centres depending on their competencies, organizational maturity, and availability of structural and technological resources
	7	Hub Centres are reference centres for the management of complex cases, as well as for clinical trials, research activities, training, second opinions, teleconsultation services and participation in regional or national clinical registries. Hub Centres provide advanced specialist support to Spoke Centres through case review, multidisciplinary discussion, and second-level assessments
	8	When formally authorized, Hub Centres are responsible for the prescription and management of anti-amyloid therapies and must ensure access to validated biomarkers (CSF/PET/plasma), apolipoprotein E (APOE) genotyping, expert neuroradiology for ARIA monitoring, a dedicated infusion setting, and structured multidisciplinary expertise
	9	Spoke Centres are Centres for Cognitive Disorders and Dementias (CCDDs), located in community or hospital settings and equipped with structured neurological and/or geriatric expertise, clinical neuropsychology, access to basic neuroimaging, and the capacity for longitudinal patient management
	10	Spoke Centres are responsible for clinical diagnosis, definition of the therapeutic plan, prescription of symptomatic treatments, certifications and exemptions, counselling, follow-up, and selection of patients to be referred to Hub Centres for advanced biological investigations and assessment of eligibility for disease-modifying therapies (DMTs)
	11	Spoke Centres must adopt a structured assessment plan for patients potentially eligible for anti-amyloid therapies, including at least: • comprehensive clinical assessment, including comorbidities, frailty, and ongoing therapies; • standardized cognitive assessment using global scales and domain-specific tools; • functional and psychological/psychiatric assessment, including autonomy, behavioural symptoms, and family context; • administration of Patient-Reported Outcome Measures (PROMs) and Patient-Reported Experience Measures (PREMs); • brain MRI with appropriate protocols, including T2*/Susceptibility-Weighted Imaging (SWI) and Fluid-Attenuated Inversion Recovery (FLAIR); • access to CSF or plasma biomarkers, when indicated and available, particularly in cases of suspected early-onset Alzheimer’s disease Based on these elements, the Spoke Centre prepares a justified referral to the Hub Centre, including all relevant clinical and instrumental documentation
	12	Spoke Centres retain responsibility for the longitudinal clinical management of patients with mild cognitive impairment (MCI) and dementia, regardless of eligibility for disease-modifying therapies (DMTs). This responsibility includes clinical diagnosis, prescription and monitoring of symptomatic treatments, counselling, certifications, documentation for exemptions and disability status, caregiver support, definition of follow-up, and coordination with GPs, Multidisciplinary Assessment Units (UVMs), and social services
	13	According to DM77, Multidimensional Assessment Units (UVMs) constitute a territorial multidisciplinary assessment function within the network and should be activated for patients with cognitive disorders associated with frailty, disability, multimorbidity, social vulnerability or complex care needs. They support the definition of personalised care plans and ensure integration between primary care, specialist services, social care and community-based resources
	14	Multidimensional assessment should not be limited to the frail elderly population but should also include complex non‑elderly neurological patients, in whom disability, cognitive impairment, behavioral disturbances, neurological comorbidities, and social vulnerability may generate care needs comparable to or even greater than those of the geriatric population
	15	UVMs should include or have formal access to GP, neurologists, geriatricians, nurses, psychologists, social workers and, where appropriate, rehabilitation and palliative care professionals. Neurological expertise is required for patients with atypical cognitive syndromes, early-onset disease, neurological comorbidities, disability, behavioural-neurological syndromes, or complex non-geriatric frailty
	16	UVMs are located within highly accessible territorial settings, such as Community Health Centres (*Case di Comunità*) or equivalent district-level facilities
	17	Diagnosis follows an integrated pathway: GPs identify cases requiring further assessment; Spoke Centres perform structured multidimensional evaluation, including brain MRI and, when indicated and available, CSF and/or plasma biomarkers; selected cases are referred to Hub Centres, where documentation is reviewed, additional assessments may be performed, and cases are discussed by a multidisciplinary team for diagnostic definition and treatment eligibility assessment
	18	The regional network adopts a telemedicine model and an integrated, interoperable information infrastructure to reduce patient mobility and optimize monitoring, including tele-visits and tele-consultations among territorial services, Spoke Centres, and Hub Centres
	19	Regional governance ensures structured monitoring of the network through KPIs defined within the ICP, which may be subject to audit to identify areas for improvement and implement corrective or training interventions
	20	The network model must be flexible and adaptable to different regional contexts, allowing modulation of the distribution and location of UVMs, Spoke Centres, and Hub Centres based on available facilities, population density, and local epidemiological characteristics
	21	The network is conceived as a dynamic and evolving model, in which activities are progressively reallocated from higher-specialization levels to community-based levels through strengthening of local competencies, structured collaboration among care levels, and use of telemedicine

**Table 2:** primary care, role of neurology, role of neuropsychology, role of neuroradiology, biomarkers, role ofgeriatrics, infusion centres and DMT treatment, communication with patients and caregivers statements

Domain	Statement *N*	Statement
Primary Care	22	The GP represents the first point of identification of cognitive and functional needs, through the recognition of cognitive complaints reported by the patient or caregiver, collection of medical history, assessment of comorbidities, and, following appropriate training, the use of cognitive and frailty assessment tools
	23	During dedicated visits or following a caregiver’s report, the GP should use standardized tools (e.g. *GP Assessment of Cognition* GPCog, *Primary Care Frailty Index* PC‑FI). Based on the results of cognitive and frailty assessment, patients follow differentiated pathways: patients with mild frailty and mild cognitive decline should be referred to Spoke Centres for further diagnostic assessment, while patients with severe frailty and advanced dementia should be preferably referred to UVMs for comprehensive multidimensional assessment and definition of a personalised care programme
	24	Referral from the GP to a Spoke Centre must be accompanied by a complete medical history and clinical assessment, the laboratory tests required by the Integrated Care Pathway (ICP), a recent electrocardiogram (ECG), and a computed tomography (CT) scan or brain MRI
	25	Regions, in collaboration with scientific societies, promote training programs on early identification of cognitive impairment and dementia, appropriate patient management by GPs, and referral pathways within the service network. Regions also ensure timely and accurate information for GPs regarding the regional network, including the location of Hub and Spoke Centres and the availability of diagnostic resources
Role of neurology	26	Neurologists are central to the diagnostic framing and differential diagnosis of cognitive disorders, particularly in early-onset, atypical, rapidly progressive, vascular, inflammatory, epileptic, parkinsonian and mixed presentations
	27	Neurologists contribute across all levels of the network, including Spoke and Hub centres, UVMs, teleconsultation pathways and hospital-based care
	28	In Spoke centres, neurologists coordinate or contribute to the clinical assessment, treatment planning, certification, symptomatic treatment, longitudinal follow-up and selection of patients requiring Hub referral
	29	In Hub centres, neurologists contribute to biological diagnosis, eligibility assessment for DMTs, ARIA clinical monitoring, management of neurological adverse events, clinical trials and second-opinion pathways
Role of Neuropsychology	30	Cognitive assessment is an integral component of Hub and Spoke Centres activities and contributes to diagnosis through structured evaluation of cognitive, functional, and behavioural profiles, supporting clinical framing, diagnostic decisions, and identification of patients potentially eligible for anti-amyloid therapies
	31	The regional network adopts a shared set of neuropsychological tests, including screening tools for UVMs and second‑level assessments for Spoke and Hub Centres, with structured reporting to ensure comparability of results throughout the care pathway
	32	Cognitive assessment is integrated into the longitudinal monitoring of patients receiving disease-modifying therapies, through periodic reassessment of cognitive functions and functional abilities using tools with high sensitivity to change
Role of Neuroradiology	33	The regional network adopts standardized MRI protocols for diagnostic assessment, follow‑up, and emergency evaluation, defining mandatory minimum sequences and specific parameters for the detection and classification of ARIA
	34	The regional network implements a neuroradiological telereporting system, identifying Hub Centres responsible for centralized reporting of neuroradiological examinations for patients receiving disease-modifying therapies
	35	Follow-up MRIs conducted for treatment monitoring are preferably performed at the same imaging site throughout the treatment period, to allow accurate sequential comparison of images
	36	MRIs for therapeutic follow‑up are classified as dedicated procedures, distinct from diagnostic MRIs, and performed using reduced protocols to optimize examination time, imaging capacity, and resource use
Biomarkers	37	To ensure diagnostic consistency across centres, regional networks adopt standardized reporting formats for Alzheimer’s disease biomarkers (CSF, amyloid PET), including numerical values, reference cut-offs, and uniform interpretative conclusions
	38	The introduction of plasma biomarkers is planned progressively, initially within Hub Centres with diagnostic expertise and subsequently, once validated, extended to upstream care settings (e.g. Spoke Centres) to support early diagnosis and follow-up
Role of Geriatrics	39	At territorial or hospital level, geriatricians contribute to early identification of patients with suspected cognitive decline through cognitive screening and frailty assessment. In cases compatible with MCI or mild dementia and non-severe frailty, patients are referred to Spoke Centres for further diagnostic evaluation
	40	Within Multidimensional Assessment Units (UVMs), geriatricians contribute to the continuous management of patients with cognitive impairment who are not eligible for referral to Spoke Centres, by defining follow‑up plans, monitoring functional autonomy and frailty, and proposing interventions aimed at reducing or stabilising frailty and preventing secondary disability
	41	In Spoke Centres, geriatricians perform diagnostic assessments and, where appropriate, prepare a justified referral to the Hub Centre for evaluation of eligibility for disease-modifying therapies
	42	In Hub Centres, following a multidimensional geriatric assessment, geriatricians collaborate with neurologists in selecting patients eligible for disease-modifying therapies, contribute to longitudinal monitoring, assess functional outcomes, and regularly review pharmacological treatments to reduce systemic and iatrogenic complications
Infusion centres and DMT treatment	43	Regional Health Authorities, in agreement with the Ministry of Health, define clear and verifiable structural, organizational, and professional requirements for the identification of Hub Centres authorized to administer disease‑modifying therapies
	44	Centres meeting the defined requirements declare their management capacity, expressed as the number of patients treatable over a given period, based on structured planning of infusions, follow‑up visits, and MRI monitoring
	45	Regions assess the overall treatment capacity at regional level by estimating the number of centres that can be activated and the annual volume of patients that can be managed, ensuring the availability of pathways for emergency management
	46	The treatment capacity of Hub Centres represents the basis for planning the disease-modifying therapy pathway and for defining the expected volumes of eligible and treatable patients within the regional network
	47	Patients eligible for disease‑modifying therapies access the Hub Centre identified for their territorial area of residence
	48	Hub Centres establish integrated pathways with neuroradiology services, emergency departments, and inpatient wards to ensure timely management of acute events or treatment‑related complications
	49	At the end of treatment or in case of discontinuation, Hub Centres activate a structured transition pathway toward Spoke Centres, including a detailed clinical report and a shared follow‑up plan
Communication with patients and caregivers	50	Within the multidisciplinary team, psychologists play an integral role in the disease‑modifying therapy pathway, actively contributing to counselling, follow‑up, and communication with patients
	51	Patients potentially eligible for disease‑modifying therapies receive structured pre‑treatment counselling delivered by the multidisciplinary team, aimed at explaining expected benefits, risks, and monitoring requirements, and at supporting informed decision‑making
	52	Caregiver involvement is agreed with the patient, in accordance with the patient’s wishes, and may include participation in decision‑making processes and informed consent
	53	Patients and, where involved, caregivers receive validated informational materials regarding mechanism of action, potential side effects, and logistical aspects of treatment
	54	In cases of adverse events or treatment discontinuation, Hub Centres adopt structured communication protocols to ensure clear, timely, and coordinated communication by the multidisciplinary team
	55	Hub Centres provide dedicated communication channels (e.g. phone or email) to facilitate communication between patients, caregivers where involved, and the multidisciplinary team

The Network Structure domain (21 statements) defines the overall organisational framework based on a regional network model articulated across territorial services, Spoke centres, and Hub centres, covering governance, roles and referral criteria, adoption of a shared Integrated Care Pathway (ICP), coordination mechanisms, telemedicine, KPI-based monitoring, and network flexibility Table 1.

The remaining domains address: early identification and GP referral pathways (Primary Care, 4 statements); the role of neurologists across the pathway, including diagnostic framing, network coordination, patient selection, and management of advanced therapies (Role of Neurology, 4 statements); cognitive assessment in diagnosis and longitudinal monitoring (Role of Neuropsychology, 3 statements); standardised MRI protocols, ARIA monitoring, and follow-up imaging (Role of Neuroradiology, 4 statements); biomarker reporting and progressive introduction of plasma biomarkers (Biomarkers, 2 statements); the role of geriatricians across the pathway (Role of Geriatrics, 4 statements); infusion centre requirements, capacity planning, and treatment transition (Infusion Centres and DMT Treatment, 7 statements); and structured counselling, caregiver involvement, and dedicated communication channels (Communication with Patients and Caregivers, 6 statements) Table 2.

## Consensus ratings

### Overall analysis

The panel reached consensus on both appropriateness and priority of implementation for all 55 statements using the RAND/UCLA Appropriateness Method. No statement was classified as inappropriate, of low priority, or uncertain in either voting round.

Appropriateness ratings were concentrated toward the upper end of the scale: 38 statements (69.1%) received a median rating of 9, while the remaining 17 statements (30.9%) received a median rating of 8. A similar pattern was observed for priority of implementation, with 16 statements (29.1%) assigned a median rating of 9 and 39 statements (70.9%) a median rating of 8 (Supplemental Materials).

### Thematic domains analysis

Appropriateness and priority ratings were also examined across the nine thematic domains. Ratings were summarised using the median of the statement‑level medians, and variability in panel responses was described using the median statement range, defined as the difference between the maximum and minimum ratings assigned to each statement.

Median appropriateness ratings were consistently high across all domains, ranging from 8 to 9.

Variability in appropriateness ratings was generally limited, with the exception of the domain “Role of Neuropsychology”, which showed the highest variability (median range = 5), followed by “Role of Neurology” (median range = 3.5) Table 3.

**Table 3:** Appropriateness and priority of implementation ratings by thematic domain

Domain	N°Statements (%)	Appropriateness median*	Median range**	Priority of implementation median*	Median range**
Network structure	21 (38.2)	9	3	8	3
Primary care	4 (7.3)	8.5	2.5	8	3.5
Role of Neurology	4 (7.3)	8.5	3.5	8.5	3
Role of Neuropsychology	3 (5.5)	8	5	8	3
Role of Neuroradiology	4 (7.3)	9	2	8	2.5
Biomarkers	2 (3.6)	9	2	8	4
Role of Geriatrics	4 (7.3)	9	3	8	4
Infusion Centres and DMT Treatment	7 (12.7)	9	3	9	2
Communication with Patients and Caregivers	6 (10.9)	9	3	8	2,5

*Always presents as the median of the statement‑level medians

** Median of the Differences between the maximum and minimum ratings assigned to each statement of the domain

Priority of implementation ratings showed slightly greater variation between domains. The domain “Infusion Centres and DMT Treatment” received the highest median priority rating (median = 9) and showed limited variability (median range = 2). Greater variability in priority ratings was observed in the domains “Biomarkers” and “Role of Geriatrics” (median range = 4), followed by “Primary care” (median range = 3.5).

## Discussion

The consensus process, which achieved full agreement across all 55 statements, reflects the panel''s recognition of the need for structural changes and provides pragmatic recommendations to guide the reorganisation of AD care in Italy. Appropriateness ratings were concentrated at the upper end of the scale, with 38 statements (69.1%) receiving a median rating of 9 and the remaining 17 (30.9%) a median rating of 8. A broadly concordant pattern was observed for priority of implementation, though with a slightly wider distribution across domains, reflecting nuanced differences in the perceived urgency of specific organisational components.

“Network structure” was the largest thematic domain of the consensus, comprising 21 statements (38.2% of the total) and achieving a median appropriateness rating of 9 Table 3, Supplemental Materials. The endorsed statements envision a comprehensive AD care network organised at regional level, designed to support the full spectrum of care for patients with cognitive decline, from early identification and diagnosis to long-term management, including, where applicable, access to disease-modifying therapies. The proposed network also includes a dedicated regional governance body responsible for ensuring coordination across the network nodes, overseeing the definition and periodic updating of the Integrated Care Pathway (ICP), and guaranteeing structured performance monitoring. This governance body should include representatives from all professional domains involved in AD care, including neurology, geriatrics, neuroradiology, nuclear medicine, neuropsychology, and primary care, to guarantee coherence in clinical practice and reduce the heterogeneity currently observed across Italian regions [16].

**Fig. 2:**
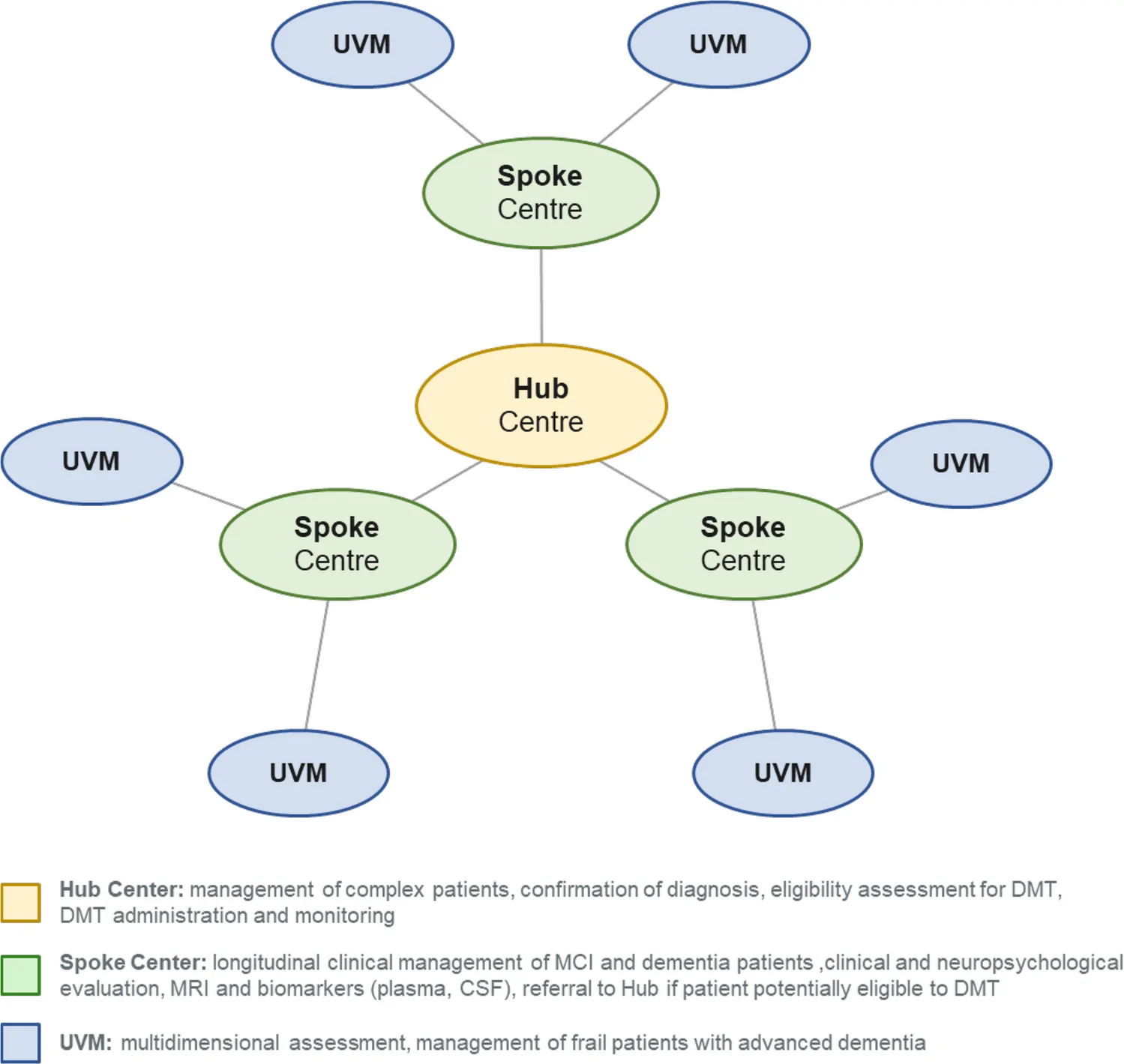
Proposed integrated care network for AD management organised across three functional levels. Abbreviations: DMT: Disease-Modifying Therapy; MRI: Magnetic Resonance Imaging; UVM: Unità di Valutazione Multidimensionale (Multidimensional Evaluation Unit)

The care network itself should be organised across three functional levels: territorial services, Spoke centres, and Hub centres, each contributing to distinct phases of the patient journey and managing different patient groups according to their clinical needs Fig. 2.

**Fig. 3:**
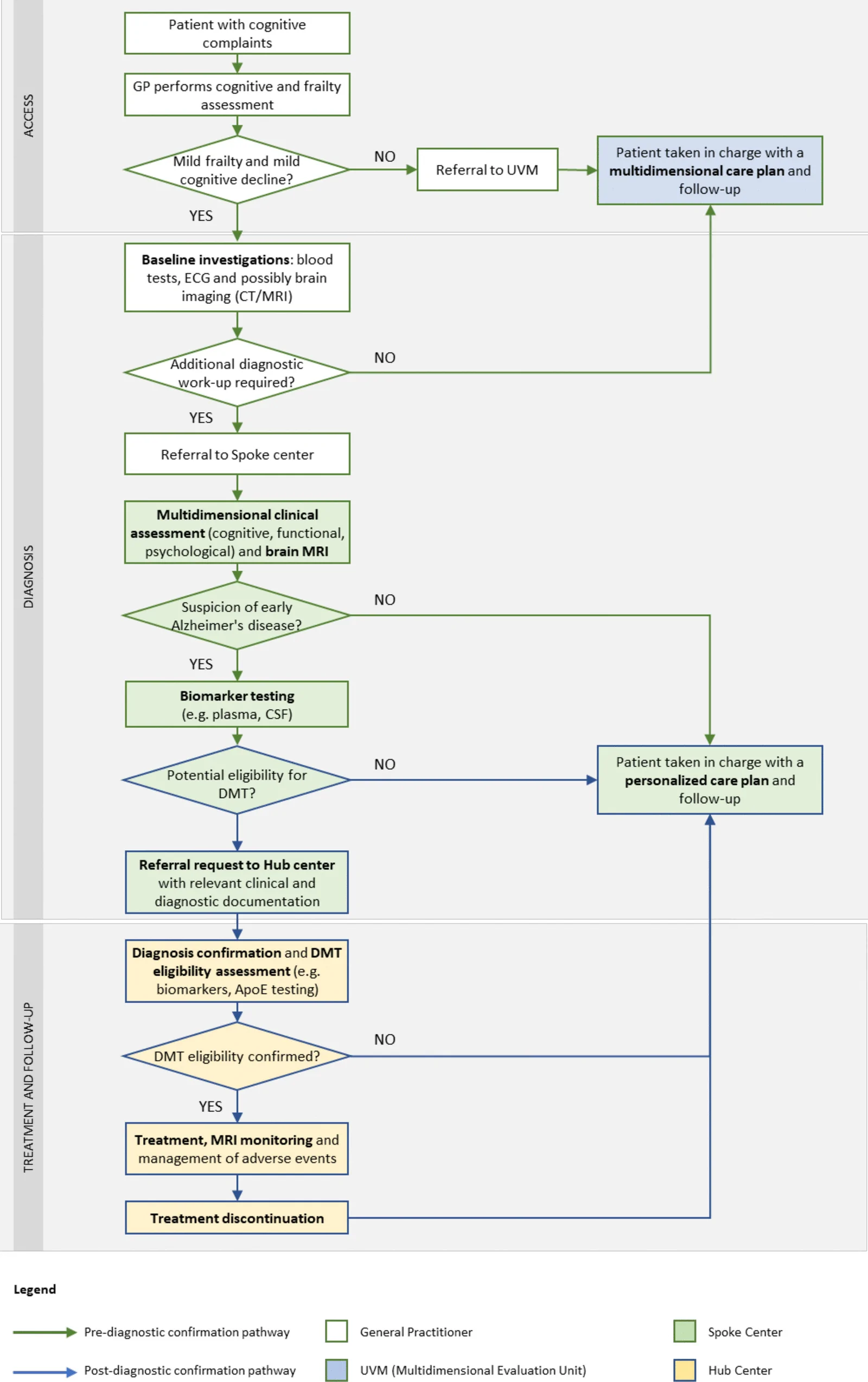
Patient pathway across the different levels of the proposed care network for AD management. GP: General Practitioner; ECG: Electrocardiogram; CT: Computed Tomography; MRI: Magnetic Resonance Imaging; CSF: Cerebrospinal Fluid; DMT: Disease-Modifying Therapy; ApoE: Apolipoprotein E

At the territorial level, GPs represent the first point of contact for individuals with suspected cognitive decline or functional impairment. Through an initial assessment incorporating cognitive and frailty screening, they direct patients towards the most appropriate referral pathway: those without severe frailty and with mild cognitive impairment are referred to Spoke centres as potential candidates for further diagnostic workup, while those presenting with severe frailty and more advanced cognitive decline are referred to Multidimensional Evaluation Units (UVM) for comprehensive assessment and care plan Fig. 3.

UVMs should not be conceived as exclusively social-care units, but as multidisciplinary bodies integrating primary care, neurology, geriatrics, nursing, psychology, and social care, in order to ensure that individuals with complex neurological needs associated with cognitive impairment, disability, or behavioural symptoms receive appropriate specialist evaluation and longitudinal care.

Spoke centres represent the second level of the proposed network Fig. 3. They should correspond to hospital- or territory-based CCDDs that meet defined organisational and clinical requirements, including neurological and geriatric expertise, neuropsychological services, and access to standard neuroimaging. They should receive patients without severe frailty and with mild cognitive impairment, identifying those who may benefit from advanced biomarker assessment. Regardless of DMT eligibility, Spoke centres retain overall responsibility for the long-term management of MCI and dementia, including diagnosis, symptomatic treatment, counselling, certification, and structured follow-up coordinating care with GPs, UVMs, and social services.

Finally, Hub centres represent the highest level of specialisation within the proposed network Fig. 3 and consist of highly specialised centres for the management of Alzheimer’s disease, including university hospitals, research hospitals, and advanced hospital-based CCDDs. They are responsible for complex case management, second opinions, clinical trials, research, and participation in clinical registries. Hub centres provide confirmatory diagnoses for patients referred from Spoke centres and conduct multidisciplinary eligibility assessments for DMTs. When formally authorised, they additionally manage the full prescription and safety monitoring cycle for anti-amyloid therapies, including structured MRI surveillance for ARIA, with the required biomarker infrastructure (CSF, PET, or plasma), APOE genotyping, and dedicated infusion capacity.

This flexible structure of the network is designed to direct each patient toward the most appropriate level of care, ensuring that specialist resources are matched to clinical complexity.

The proposed network relies on coordinated contribution of multiple professional figures.

The “Role of Neurology” domain (4 statements, median appropriateness = 8.5, median priority = 8.5; Table 3, Supplemental Materials) highlights the central role of neurologists across all network levels. The statements emphasised the importance of embedding neurological expertise within UVMs, given the central role of neurologists in the differential diagnosis of cognitive disorders. The presence of a neurologist also enables specialist reassessment and ensures appropriate redirection to Spoke centres when further diagnostic work-up is warranted. At Spoke centres, neurologists coordinate clinical assessment, treatment planning, symptomatic treatment, certification, and selection of patients for Hub referral. Finally, at Hub level, neurologists contribute to biological diagnosis, DMT eligibility assessment, ARIA clinical monitoring, adverse event management, and clinical trials.

Geriatric medicine emerged as an important contributor across all network levels (“Role of Geriatrics” domain, median appropriateness = 9, median priority = 8; Table 3, Supplemental Materials). Geriatricians apply a comprehensive, function-oriented approach: supporting early identification and frailty assessment at community level, interpreting cognitive symptoms within overall clinical complexity at Spoke centres, and contributing to multidisciplinary treatment eligibility assessment at Hub level. Geriatric expertise is equally essential for the longitudinal management of patients who are not candidates for DMTs.

The “Primary care” domain showed on of the highest within-domain variability across the consensus, with a median range of 3.5 for priority of implementation. In line with the statements, GPs are expected to act as the first point of contact, identifying cognitive complaints, assessing comorbidities, and applying standardized tools for cognitive and frailty screening. Furthermore, they are responsible for appropriately directing patients, along differentiated referral pathways, towards Spoke centres for further diagnostic assessment in cases of mild frailty and mild cognitive decline, and towards UVMs for comprehensive multidimensional assessment in cases of severe frailty and advanced dementia. The within-domain variability likely reflects the need to build the system-level conditions required to support the critical role attributed to GPs in the early identification and initial assessment of cognitive and functional impairment. Moreover, the panel recognized the need for up-to-date network information and structured training programmes promoted at regional level in collaboration with scientific societies.

The “Role of Neuropsychology” domain (3 statements, median appropriateness = 8, median priority = 8; Table 3, Supplemental Materials) recorded the widest appropriateness range across all domains (median range = 5). This could likely reflect heterogeneous availability and integration of neuropsychological services across Italian centres. The panel strongly agreed that standardised neuropsychological assessment, with harmonised test batteries, structured reporting formats, and tools sensitive to change for longitudinal monitoring, should be a core element of both diagnostic evaluation and follow-up across all network levels.

Within the broader AD care model endorsed by the panel, the organisation of DMT delivery represented the domain of strongest and most uniform consensus. The seven statements of the “Infusion Centres and DMT Treatment domain” achieved the highest median ratings for both appropriateness and priority of implementation (both median = 9), with minimal variability in panel responses (median range = 2–3) (Table 3, *Supplemental Materials*). During the initial phases of implementation, DMT-related activities are expected to be concentrated within Hub centres, which must clearly define their treatment capacity in terms of infusion slots, clinical follow-up visits, and MRI monitoring availability, while ensuring close operational integration with neuroradiology services, emergency departments, and inpatient wards to enable timely recognition and management of potential treatment-related complications. Importantly, however, as clinical experience grows and local capabilities expand, components of patient management are expected to progressively shift toward more proximal levels of care. This progressive territorialisation, supported by telemedicine (e.g. remote specialist consultations), shared clinical protocols, and ongoing professional training, is considered essential to ensure long-term sustainability and to reduce the risk of geographic inequity in access to innovative therapies.

The safe and effective delivery of DMTs places equally high demands on neuroradiology, which emerged as another area of strong and consistent consensus. Indeed the “Role of Neuroradiology” domain showed a median appropriateness rating of 9, a median priority rating of 8, and low variability across panel members (median range = 2) Table 3, Supplemental Materials. This reflects a shared recognition of the pivotal role of MRI both in the diagnostic work-up of AD, particularly for excluding alternative aetiologies, and in the detection and longitudinal monitoring of ARIA in patients undergoing treatment. The panel strongly supported the implementation of standardised MRI protocols across the care network, with mandatory sequences and harmonised acquisition parameters considered essential to optimise ARIA detection and ensure consistency in image interpretation [22]. Neuroradiological tele-reporting was also endorsed as an effective strategy to facilitate access to specialised expertise in peripheral or resource-limited settings.

A comparable need for standardisation extends to biomarker investigations, where the panel emphasised the importance of adopting uniform reporting formats, including quantitative values, reference thresholds, and clearly defined diagnostic interpretations, to promote consistency and support clinical decision-making across centres. In parallel, the panel acknowledged the emerging role of plasma biomarkers [23, 24], envisioning a phased introduction beginning within specialised centres and potentially extending to earlier stages of the diagnostic pathway as validation and regulatory frameworks mature. Standardisation is also being pursued at European level: the European intersocietal recommendations define a stepwise, patient-centred diagnostic workflow based on first- and, where needed, second-line biomarker testing [25], and the present statements are consistent with that framework. The two documents operate at complementary levels: the European recommendations define which biomarker is appropriate for which patient, while the present statements address how that pathway can be delivered within a regionally organised health system.

The proposed network model addresses the full continuum of AD care, encompassing early identification, accurate diagnosis, appropriate triage, longitudinal management of patients with MCI and dementia regardless of treatment eligibility, caregiver support, and the progressive adoption of innovative technologies. DMTs represent a major catalyst for network redesign in the current period, but CCDDs must continue to fulfil their broader mandate for all patients with cognitive disorders, including those who will never be candidates for disease-modifying treatment.

Taken together, the high level of agreement achieved in this consensus, combined with a careful reading of where variability remains, offers both a clear direction for policy and a realistic acknowledgement of the implementation challenges ahead.

Finally, the RAPID project establishes a multi-level organizational network that could be leveraged in the future to support the identification and referral of individuals at very early stages of disease, including those with subjective cognitive decline, as advances in blood-based biomarkers increasingly enable biology-based identification before the onset of measurable cognitive deficits [26]. Furthermore, its flexible structure may facilitate the future integration of emerging diagnostic and therapeutic innovations as their clinical utility becomes more clearly established [27–29].

## Conclusion

The consensus developed within the RAPID project, which achieved full agreement across all 55 statements, envisions a regional network structured across three complementary levels, territorial services, Spoke centres, and Hub centres, coordinated by a dedicated regional governance body.

This structure is designed to ensure that AD patients are assigned to the most appropriate level of care, optimising each component of the diagnostic and therapeutic pathway and the use of specialist resources while preserving equitable access to the latest treatment advancements across different regional contexts.

Translating these recommendations into practice will require coordinated action at both national and regional levels, including the formal designation of Hub and Spoke centres, the development and adoption of standardised ICPs, and the progressive strengthening of territorial services.

The RAPID recommendations are intended as a practical reference for regional health authorities and policymakers engaged in planning the reorganisation of AD care in Italy, with the broader aim of ensuring that the system is prepared to meet the evolving needs of people living with Alzheimer’s disease and their caregivers, including readiness for therapeutic innovation.

### Limitation

Although the RAND/UCLA Appropriateness Method provides a validated consensus framework, the findings are based on expert judgement and are subject to a degree of subjectivity. Variability in specific domains likely reflects heterogeneity in current practice and organisational readiness rather than disagreement on clinical principles, and may affect implementation feasibility and timing. Health economic evaluation and prospective validation in real-world settings were beyond the scope of this work and should be addressed in future research.

## Supplementary Information

Below is the link to the electronic supplementary material.

Supplementary file1 (DOCX 39 KB)
