# Supplementary material for: Organizing healthcare delivery in the era of disease-modifying therapies for Alzheimer’s Disease: an Italian RAND/UCLA consensus
Source: Neurol Sci. 2026 Oct 9;47(11):850. doi: 10.1007/s10072-026-09452-z (PMC13649915; doi:10.1007/s10072-026-09452-z)
Supplement: Supplementary file 1 [file 10072_2026_9452_MOESM1_ESM.docx]

***Supplemental materials***

Panel ratings for all 55 consensus statements across the nine thematic domains. For each statement, the median score, minimum and maximum ratings, median range are reported separately for appropriateness and priority of implementation

|  |  | **Appropriateness** | | | | **Priority of implementation** | | | | |
| --- | --- | --- | --- | --- | --- | --- | --- | --- | --- | --- |
| **Domain** | **Statement N.** | **Median** | **Min** | **Max** | **∆**  [max-min] | | **Median** | **Min** | **Max** | **∆**  [max-min] |
| **Network structure** | **1** | 9 | 7 | 9 | 2 | | 9 | 7 | 9 | 2 |
|  | **2** | 9 | 6 | 9 | 3 | | 9 | 6 | 9 | 3 |
|  | **3** | 9 | 6 | 9 | 3 | | 8 | 7 | 9 | 2 |
|  | **4** | 9 | 6 | 9 | 3 | | 9 | 7 | 9 | 2 |
|  | **5** | 9 | 6 | 9 | 3 | | 8 | 6 | 9 | 3 |
|  | **6** | 9 | 7 | 9 | 2 | | 8 | 6 | 9 | 3 |
|  | **7** | 9 | 8 | 9 | 1 | | 8 | 8 | 9 | 1 |
|  | **8** | 9 | 7 | 9 | 2 | | 9 | 8 | 9 | 1 |
|  | **9** | 9 | 7 | 9 | 2 | | 8 | 7 | 9 | 2 |
|  | **10** | 8 | 6 | 9 | 3 | | 8 | 7 | 9 | 2 |
|  | **11** | 8 | 6 | 9 | 3 | | 8 | 7 | 9 | 2 |
|  | **12** | 8 | 7 | 9 | 2 | | 8 | 6 | 9 | 3 |
|  | **13** | 8 | 6 | 9 | 3 | | 8 | 6 | 9 | 3 |
|  | **14** | 8 | 6 | 9 | 3 | | 8 | 6 | 9 | 3 |
|  | **15** | 8 | 6 | 9 | 3 | | 8 | 6 | 9 | 3 |
|  | **16** | 8 | 7 | 9 | 2 | | 8 | 6 | 9 | 3 |
|  | **17** | 8 | 3 | 9 | 6 | | 9 | 6 | 9 | 3 |
|  | **18** | 9 | 6 | 9 | 3 | | 9 | 5 | 9 | 4 |
|  | **19** | 8 | 7 | 9 | 2 | | 8 | 5 | 9 | 4 |
|  | **20** | 9 | 6 | 9 | 3 | | 8 | 6 | 9 | 3 |
|  | **21** | 8 | 5 | 9 | 4 | | 8 | 5 | 9 | 4 |
| **Primary Care** | **22** | 8 | 6 | 9 | 3 | | 8 | 6 | 9 | 3 |
|  | **23** | 9 | 7 | 9 | 2 | | 9 | 6 | 9 | 3 |
|  | **24** | 8 | 6 | 9 | 3 | | 8 | 5 | 9 | 4 |
|  | **25** | 9 | 8 | 9 | 1 | | 8 | 5 | 9 | 4 |
| **Role of Neurology** | **26** | 9 | 6 | 9 | 3 | | 9 | 4 | 9 | 5 |
|  | **27** | 8 | 6 | 9 | 3 | | 8 | 7 | 9 | 2 |
|  | **28** | 8 | 5 | 9 | 4 | | 8 | 5 | 9 | 4 |
|  | **29** | 9 | 5 | 9 | 4 | | 9 | 8 | 9 | 1 |
| **Role of Neuropsychology** | **30** | 9 | 4 | 9 | 5 | | 8 | 6 | 9 | 3 |
|  | **31** | 8 | 6 | 9 | 3 | | 8 | 6 | 9 | 3 |
|  | **32** | 8 | 4 | 9 | 5 | | 8 | 6 | 9 | 3 |
| **Role of Neuroradiology** | **33** | 9 | 7 | 9 | 2 | | 9 | 7 | 9 | 2 |
|  | **34** | 9 | 7 | 9 | 2 | | 8 | 6 | 9 | 3 |
|  | **35** | 9 | 7 | 9 | 2 | | 8 | 7 | 9 | 2 |
|  | **36** | 9 | 7 | 9 | 2 | | 8 | 6 | 9 | 3 |
| **Biomarkers** | **37** | 9 | 7 | 9 | 2 | | 8 | 6 | 9 | 3 |
|  | **38** | 9 | 7 | 9 | 2 | | 8 | 4 | 9 | 5 |
| **Role of Geriatrics** | **39** | 9 | 6 | 9 | 3 | | 8 | 6 | 9 | 3 |
|  | **40** | 9 | 6 | 9 | 3 | | 8 | 5 | 9 | 4 |
|  | **41** | 9 | 6 | 9 | 3 | | 9 | 5 | 9 | 4 |
|  | **42** | 9 | 6 | 9 | 3 | | 8 | 5 | 9 | 4 |
| **Infusion centres and DMT treatment** | **43** | 9 | 7 | 9 | 2 | | 9 | 7 | 9 | 2 |
|  | **44** | 9 | 7 | 9 | 2 | | 9 | 7 | 9 | 2 |
|  | **45** | 9 | 6 | 9 | 3 | | 9 | 7 | 9 | 2 |
|  | **46** | 9 | 6 | 9 | 3 | | 8 | 7 | 9 | 2 |
|  | **47** | 8 | 5 | 9 | 4 | | 8 | 6 | 9 | 3 |
|  | **48** | 9 | 6 | 9 | 3 | | 9 | 7 | 9 | 2 |
|  | **49** | 9 | 6 | 9 | 3 | | 8 | 7 | 9 | 2 |
| **Communication with patients and caregivers** | **50** | 9 | 6 | 9 | 3 | | 8 | 3 | 9 | 6 |
|  | **51** | 9 | 6 | 9 | 3 | | 8 | 5 | 9 | 4 |
|  | **52** | 9 | 6 | 9 | 3 | | 8 | 7 | 9 | 2 |
|  | **53** | 9 | 6 | 9 | 3 | | 8 | 6 | 9 | 3 |
|  | **54** | 9 | 6 | 9 | 3 | | 9 | 7 | 9 | 2 |
|  | **55** | 9 | 7 | 9 | 2 | | 8 | 7 | 9 | 2 |
